# Mialostatin, a Novel Midgut Cystatin from *Ixodes ricinus* Ticks: Crystal Structure and Regulation of Host Blood Digestion

**DOI:** 10.3390/ijms22105371

**Published:** 2021-05-20

**Authors:** Jan Kotál, Michal Buša, Veronika Urbanová, Pavlína Řezáčová, Jindřich Chmelař, Helena Langhansová, Daniel Sojka, Michael Mareš, Michail Kotsyfakis

**Affiliations:** 1 Institute of Parasitology, Biology Centre, Academy of Sciences of the Czech Republic, Branišovská 1160/31, 37005 České Budějovice, Czech Republic; jankotal@gmail.com (J.K.); veronika@paru.cas.cz (V.U.); sojka@paru.cas.cz (D.S.); 2 Department of Medical Biology, Faculty of Science, University of South Bohemia in České Budějovice, Branišovská 1760c, 37005 České Budějovice, Czech Republic; chmelar@prf.jcu.cz (J.C.); hlanghansova@prf.jcu.cz (H.L.); 3 Institute of Organic Chemistry and Biochemistry, Academy of Sciences of the Czech Republic, Flemingovo n. 2, 16610 Praha, Czech Republic; michal.busa@uochb.cas.cz (M.B.); rezacova@img.cas.cz (P.Ř.); 4 Department of Biochemistry, Faculty of Science, Charles University, Hlavova 2030/8, 12800 Prague, Czech Republic

**Keywords:** cathepsin, crystal structure, cysteine protease, digestion, *Ixodes ricinus*, midgut, parasite

## Abstract

The hard tick *Ixodes ricinus* is a vector of Lyme disease and tick-borne encephalitis. Host blood protein digestion, essential for tick development and reproduction, occurs in tick midgut digestive cells driven by cathepsin proteases. Little is known about the regulation of the digestive proteolytic machinery of *I. ricinus*. Here we characterize a novel cystatin-type protease inhibitor, mialostatin, from the *I. ricinus* midgut. Blood feeding rapidly induced mialostatin expression in the gut, which continued after tick detachment. Recombinant mialostatin inhibited a number of *I. ricinus* digestive cysteine cathepsins, with the greatest potency observed against cathepsin L isoforms, with which it co-localized in midgut digestive cells. The crystal structure of mialostatin was determined at 1.55 Å to explain its unique inhibitory specificity. Finally, mialostatin effectively blocked in vitro proteolysis of blood proteins by midgut cysteine cathepsins. Mialostatin is likely to be involved in the regulation of gut-associated proteolytic pathways, making midgut cystatins promising targets for tick control strategies.

## 1. Introduction

Ticks are globally distributed ectoparasitic arthropods that strictly feed on host blood. While soft ticks (family Argasidae) feed only for a few hours, hard ticks (family Ixodidae) usually attach to their hosts for several days to fully engorge and proceed to their next developmental stage. The hard tick *Ixodes ricinus* is found mainly in Europe but also in neighboring parts of Africa and the Middle East, where it is a major vector of pathogens such as Lyme disease spirochetes (*Borrelia burgdorferi sensu lato*), tick-borne encephalitis virus [1] or *Babesia* spp. [2]. Adult *I. ricinus* females feed for 6–9 days on a vertebrate host to enlarge over 100 times in weight [3].

Since blood is a highly specific and sole source of nutrients for these ticks, they have adapted to efficiently process large amounts of host blood. Blood degrades in the acidic endolysosomes of digestive cells of the tick midgut. Gut lumen uptake of the two main blood constituents, albumin and hemoglobin, is facilitated by two different mechanisms [4]: albumin is taken up non-specifically by fluid-phase endocytosis, while hemoglobin is recognized by specific receptor-mediated endocytosis. Subsequently, albumin is directed to small acidic vesicles and hemoglobin to a population of large digestive vesicles [4]. Despite these differences, both albumin and hemoglobin are cleaved and processed to single amino acids and short peptides by the same proteolytic system [5,6]. The degradation pathway for hemoglobin is described in detail elsewhere [4]. Briefly, the initial phase is catalyzed by three *I. ricinus* digestive endopeptidases at low pH (3.5 to 4.5) including a cysteine protease legumain (IrAE) [7] and aspartic protease cathepsin D (IrCD1) [8]. Two cysteine protease cathepsin L isoforms, IrCL1 [9] (GenBank: EF428205) and IrCL3 (GenBank: QBK51063), complement the initial phase: IrCL1 expression in tick gut cells peaks at the end of tick feeding [9], while its ortholog, IrCL3, is present in the tick midgut predominantly after feeding, where it complements the activity of IrCL1 (D. Sojka, personal communication, December 2020). Cysteine proteases with exopeptidase activity, cathepsins B and C (IrCB and IrCC), continue hemoglobin degradation to dipeptides at an optimal pH of 5.5–6.0 in digestive cells [6,10,11]. Digestion to single amino acids is facilitated by carboxypeptidase and leucine aminopeptidase [6]. Blood processing by ticks and the roles of individual proteases are reviewed in detail elsewhere [12,13].

Under physiological conditions, cysteine protease activity is regulated by proteinaceous inhibitors, including those in the cystatin family [14,15]. Cystatins are tight binding, reversible inhibitors of legumain and papain-like cysteine proteases [16]. According to MEROPS nomenclature, cystatins are subdivided into three subfamilies: I25A (type 1, stefins), I25B (type 2 and type 3, kininogens), and I25C (type 4, fetuins) [17]. Only type 1 and 2 cystatins have so far been identified in ticks [18]. Cystatins are mostly associated with the regulation of proteases involved in blood digestion and heme detoxification in the tick midgut [18] and with the modulation of the host immune system as components of tick saliva [19,20], although they have also been detected in other tick tissues [21,22].

In soft ticks, only two midgut cystatins have been functionally characterized: Om-cystatins 1 and 2 from *Ornithodoros moubata* [23]. While Om-cystatin 1 is exclusively expressed in the midgut, Om-cystatin 2 can be found in all tissues and has immunomodulatory properties when secreted into the host [24,25]. Both inhibit cathepsins B, C, and H and are involved in blood processing [23]. Gut-associated cystatins from only two *Ixodes* species have been reported to date: a gut-secreted cystatin JpIocys2a from *Ixodes ovatus* was shown to inhibit cathepsins B, C, and L [26], while the expression of three *Ixodes persulcatus* cystatins, JpIpcys2a, b, and c, was demonstrated in almost all tissues and instars [27].

Despite the relatively good characterization of the digestive proteases present in the *I. ricinus* midgut [13], there has been little functional characterization of their inhibitors and regulatory mechanisms. Here we report a novel cystatin from the *I. ricinus* midgut, mialostatin, and present its crystal structure, inhibitory specificity, tissue localization, and role in the regulation of blood digestion.

## 2. Results

### 2.1. Mialostatin Transcript Predominantly Accumulates in the Tick Midgut

In order to clone mialostatin, we used primers based on available cystatin sequences identified in *Ixodes scapularis* tick genome. To obtain the longest possible reads, we also focused on the 5’ UTRs and 3’ UTRs regions. In the course of our study, an *I. ricinus* transcriptome was published with a transcript of an identical sequence to mialostatin (Genbank accession number GFVZ01041806.1) [28]. However, since this particular transcript was obtained from whole body tick sequencing, we used BLAST to search for highly similar sequences in other transcriptomic studies to specifically localize its expression (https://blast.ncbi.nlm.nih.gov/Blast.cgi, accessed on 20 May 2021). As a result we found a highly similar transcript SigP-158801 upregulated mainly in the tick midgut [29]; similarly to another transcript GCJO01026918.1 identified in a study focusing on the tick gut [30]. To verify the localization of mialostatin, we examined its expression in different tick tissues and feeding stages and confirmed its predominantly midgut expression.

**Figure 1:**
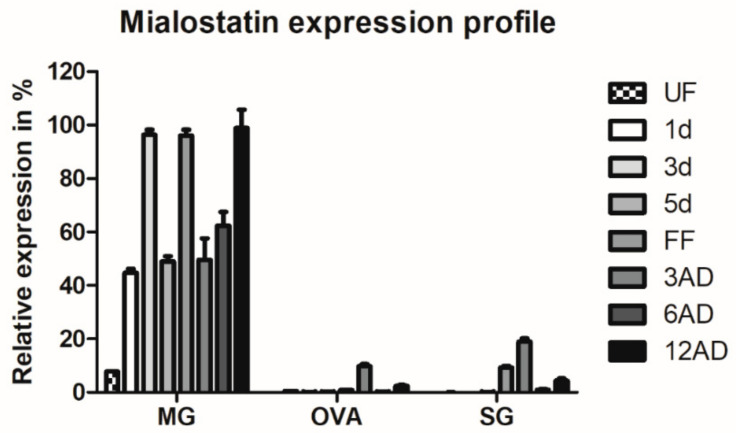
Mialostatin is predominantly produced in the tick midgut and its expression is upregulated by tick feeding. Expression maxima are prior to rapid engorgement, in fully fed females, and at two weeks post tick detachment from the host. Mialostatin expression was determined by quantitative PCR using cDNA templates prepared from a pool of three tissues from female ticks (MG—midgut, OVA—ovaries, SG—salivary glands). The qPCR output was normalized to the *I. ricinus* elongation factor 1 gene and compared across all values with the highest expression set to 100%. Data show an average of three biological replicates ± SEM. Categories: UF—unfed ticks; 1d, 3d, 5d—ticks after 1, 3, or 5 days of feeding; FF—fully fed ticks after 7–8 days of feeding; 3AD, 6AD, 12AD—ticks 3, 6, or 12 days after detachment.

Increased transcription of mialostatin over the feeding course implies an important role in tick metabolism [18]. Figure 1 shows the expression of mialostatin in the tick midgut, ovaries, and salivary glands before, during, and after feeding. Mialostatin transcript was predominantly present in the tick midgut, where expression oscillated throughout feeding and after detachment but at consistently higher expression than the other examined tissues. The presence and upregulation of mialostatin transcript in tick salivary glands and ovaries were low, peaking in fully fed ticks and at the early phase of detachment at maximum levels of only 10–20% of midgut expression (Figure 1).

### 2.2. Mialostatin Is a Broad-Spectrum Inhibitor of Cysteine Cathepsins and Is Highly Effective against Cathepsin L

Purified recombinant mialostatin was screened in vitro for its inhibitory potential against major endogenous digestive proteases present in the *I. ricinus* gut [6]. These proteases were tested in the form of recombinant enzymes or proteolytic activities in the gut tissue extract (Table 1, left and middle panels). The strongest inhibition was found for recombinant *I. ricinus* cathepsins L1 and L3 (IC_50_s of 0.071 and 0.39 nM, respectively), which are papain-type cysteine proteases and consistent with sub-nanomolar inhibition of cathepsin L-like activity by the extract (IC_50_ of 0.18 nM). The cathepsin B-like and cathepsin C-like activities of the extract were inhibited with lower potency, with IC_50_ values in the double-digit nanomolar range (12.1 and 91.7 nM, respectively). Mialostatin did not inhibit *I. ricinus* digestive proteases out of the papain family, including the aspartic protease cathepsin D1 and the clan CD cysteine protease legumain (asparaginyl endopeptidase).

We next expanded a spectrum of papain-type cysteine proteases and screened mialostatin against a representative panel of human cathepsins selected to cover a wide range of endo- and exopeptidase activities (Table 1, right panel) including endopeptidases cathepsins L, K, and S and exopeptidases cathepsin B (a peptidyl dipeptidase and endopeptidase), cathepsin C (a dipeptidyl peptidase), and cathepsin H (an aminopeptidase). Human cathepsin L was inhibited at subnanomolar concentrations (IC_50_ 0.38 nM), similar to its *I. ricinus* homologs, and all other human cysteine cathepsins were inhibited with IC_50_ values in a narrow range from 2.2 to 24 nM.

In conclusion, mialostatin displays an unusually broad inhibitory specificity against cysteine cathepsins, with a particularly strong interaction with cathepsin L isoforms. The high affinity for cysteine cathepsins with endopeptidase and exopeptidase activities clearly distinguishes mialostatin from other described *Ixodes* cystatins, which display weak or no inhibition of these exopeptidases (see below).

### 2.3. Mialostatin Is Present in the Tick Gut Wall and Lumen

We further investigated mialostatin’s distribution within the tick midgut. Gut epithelia and lumina were collected from fully fed *I. ricinus* adult females and subjected to proteomic analysis to directly determine the presence or absence of mialostatin. The LC-MS/MS strategy was based on the enzymatic digestion of a complex protein mixture and MS/MS peptide sequencing. This analysis provided 11–71% peptide coverage of the mialostatin sequence and a mass accuracy of <5 p.p.m. (Table S1), allowing us to conclude that mialostatin is present in both the gut tissue and luminal contents of *I. ricinus* ticks.

**Figure 2:**
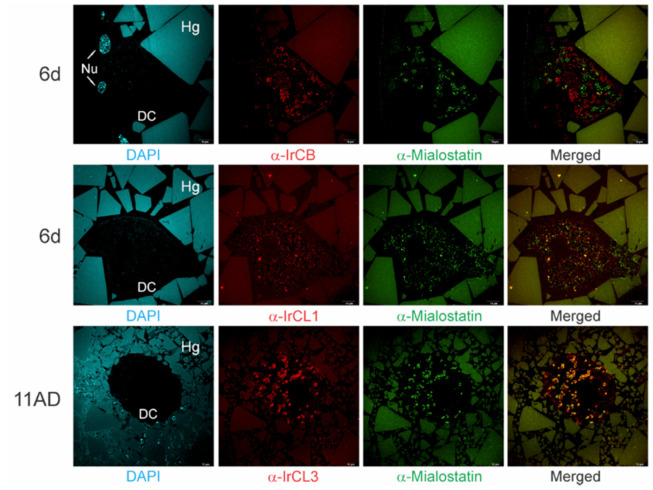
Mialostatin co-localizes with IrCL3 inside gut digestive cells of female *I. ricinus* ticks. Multicolored confocal immunofluorescence indicates variable colocalization of mialostatin (green signal) with the cathepsin-type proteases IrCB, IrCL1, and IrCL3 (red signal) in female tick gut sections at the sixth day of feeding (6d) and eleventh day post detachment from the host (11AD). Mialostatin and IrCL3 show the greatest co-localization (yellow signal in merged images), thus IrCL3 represents the most probable target protease. DAPI counterstaining is shown in cyan. DC—digestive cells; Nu—nucleus; Hg—hemoglobin crystals in gut lumen, scale bar-10 µm.

Immunolabeling was used to evaluate the potential biological selectivity of mialostatin towards different papain-like enzymes present in tick gut tissue. Localization of mialostatin with IrCB, IrCL1, and IrCL3 was examined using multicolor immunohistochemistry (Figure 2), with the sample collection and section preparation time points selected based on qPCR-determined dynamic expression profiles of individual proteases (sixth day of feeding for IrCLB and IrCL1; eleventh day post feeding for IrCL3) to establish the availability of these proteases for co-localization with mialostatin at these timepoints. IrCL3 was the most probable target protease for mialostatin, as co-localization signals at the surface of large vesicles in tick gut cells (specific ring patterns) was nearly complete. However, there was also some co-localization of mialostatin with IrCL1 but not the cysteine protease cathepsin B (IrCB).

**Figure 3:**
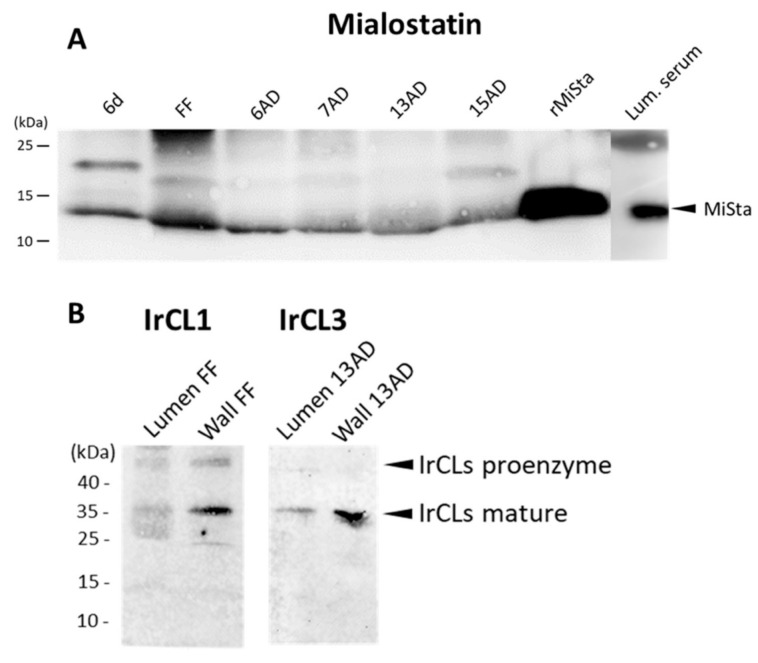
Western blot analysis of mialostatin, IrCL1, and IrCL3 in the tick midgut. (**A**) Tick midgut wall tissue extracts from various stages of tick feeding and midgut lumina from fully fed ticks were analyzed by SDS-PAGE and Western blotting. Mialostatin was labeled with a mouse monoclonal antibody and its signal detected using a fluorescently labeled secondary antibody. (**B**) Tick midgut wall and midgut lumen homogenates from fully fed ticks were analyzed by SDS-PAGE and immunoblotting with α-IrCL1 and α-IrCL3 rabbit polyclonal antibodies. Goat α-rabbit IgG Alexa 488 fluorescent secondary antibody was used to visualize protein bands using ChemiDoc MP imager. 6d—sixth day of feeding; FF—fully fed; 6, 7, 13, 15 AD—days post detachment from the host. Lum. serum—luminal fluid from ticks fed on erythrocyte-free serum; rMista—recombinant mialostatin. Full view of presented Western blots can be found in the Supplementary Materials, Figures S2–S4.

Immunoblot analyses of tick gut tissue were performed to (i) confirm mialostatin selectivity for IrCL3 and further evaluate potential interactions with IrCL1; and (ii) evaluate potential secretion of mialostatin and the cathepsin-L-like tick proteases into the gut lumen. The latter could not be observed by immunohistochemical labeling due to the rapid dilution of gut epithelial cell secretions with the large amount of imbibed host blood in the lumen. However, mialostatin was detected in the gut wall (Figure 3A) at all collected time-points during feeding. Gut tissue originating from ticks membrane-fed on pure bovine blood serum (without erythrocytes) [31] was used to avoid interference between mialostatin-specific signals and host hemoglobin proteins of identical molecular weight. IrCL1 and IrCL3 signals were also detected in both the gut epithelium (cell wall) and the gut lumen (Figure 3B). The multiple IrCL1 bands corresponded to the proenzyme and mature enzyme forms [9].

### 2.4. Mialostatin Inhibits Blood-Protein Digestion Catalyzed by Tick Gut Cysteine Cathepsins

**Figure 4:**
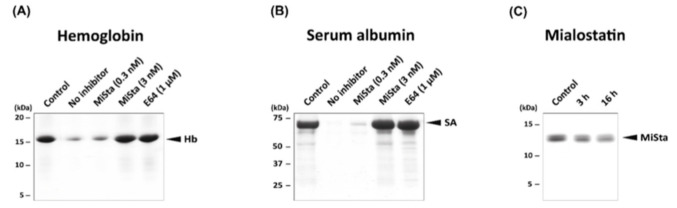
Blood protein digestion with tick gut proteases is inhibited by mialostatin. Hemoglobin (**A**) and serum albumin (**B**) were digested in vitro with *I. ricinus* gut tissue extracts in the presence and absence of mialostatin. Blood protein substrate (5 µg of hemoglobin or 10 µg of serum albumin) was incubated with 0.4 µg gut tissue extract of cysteine proteases at pH 3.6 for 16 h. The extract was pre-incubated with mialostatin (MiSta) or the general cysteine protease inhibitor E-64 at the indicated concentrations prior to initiation of digestion. The digests were subjected to Tricine-SDS-PAGE (**A**) or Laemmli-SDS-PAGE (**B**) and visualized by protein staining. The hemoglobin (Hb) and serum albumin (SA) substrates are marked; the non-digested control is indicated. (**C**) Proteolytic stability of mialostatin in the gut tissue extract. Mialostatin (5 µg) was incubated with 0.4 µg gut extract protein under the same conditions as in (**A**,**B**), subjected to Tricine-SDS-PAGE, and visualized by protein staining. Mialostatin (MiSta) is marked; the non-digested control is indicated.

In tick gut tissue, cysteine cathepsins play a critical role in the acidic degradation of the two most abundant host blood proteins, hemoglobin and serum albumin. In particular, cathepsin L is involved in the initial phase of the degradation pathway, which is continued by the action of cathepsins B and C [5,6]. We evaluated the effect of mialostatin on the in vitro degradation of hemoglobin and serum albumin by the proteolytically active extract of *I. ricinus* gut tissue (limited to cysteine proteases by treatment with inhibitors of other protease classes). Both blood proteins were digested at optimal acidic pH, and SDS-PAGE analysis demonstrated highly efficient degradation of these substrates (Figure 4A,B). These processes were effectively blocked by mialostatin in a dose-dependent manner, with complete inhibition at a low nanomolar concentration of mialostatin. A similar effect was achieved by adding E-64, a small molecule general inhibitor of cysteine cathepsins. Further, we tested the stability of mialostatin exposed to the complex proteolytic environment of the gut tissue extract (Figure 4C), which revealed that mialostatin was generally stable and showed only partial degradation over long-term treatment. In summary, the blood protein digestion catalyzed by cysteine cathepsins of *I. ricinus* can be effectively controlled by mialostatin under native-like conditions.

### 2.5. Phylogenetic Analysis and Three-Dimensional Structure of Mialostatin and Its Reactive Site

**Figure 5:**
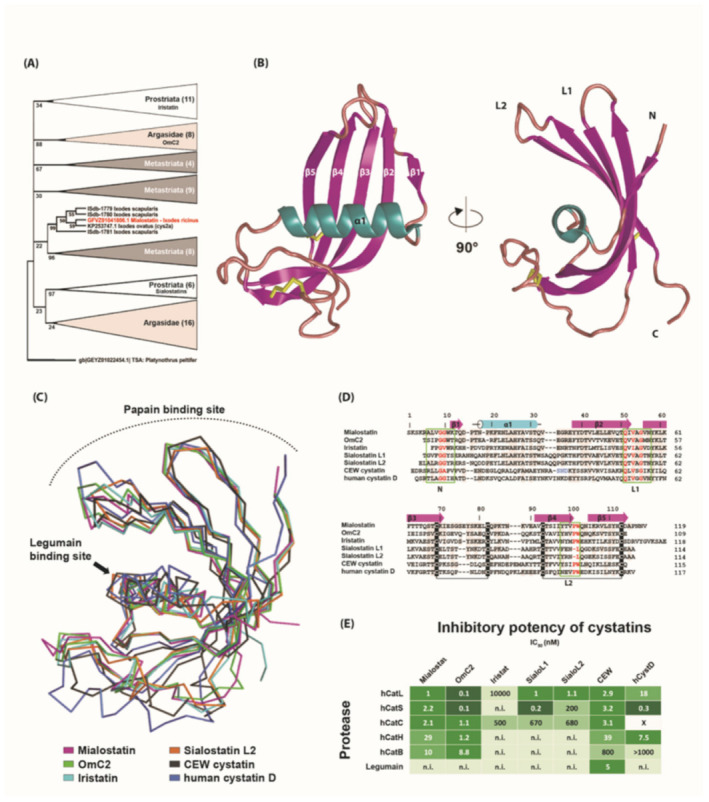
Crystal structure of mialostatin and its comparison with other family 2 cystatins. (**A**) Molecular phylogenetic analysis (maximum likelihood model) of secreted tick cystatins. Simplified consensus tree based on the maximum likelihood method with 1000-repeat bootstrap support. All clades except the one with mialostatin (highlighted in red) are condensed. Cystatin from the mite *Platynothrus peltifer* was used as an outgroup. The tree with the highest log-likelihood (−4870, 9711) is shown. Branches corresponding to partitions reproduced in less than 20% bootstrap replicates are collapsed. Numbers next to branches represent percentage of trees, in which the associated taxa clustered together during bootstrap analysis. For full tree see Figure S1. (**B**) The three-dimensional structure of mialostatin (PDB code 6ZTK) is shown as a cartoon representation colored by secondary structural elements (α1—cyan; β1-5—magenta). The N- and C-termini and two disulfide bridges, Cys69–Cys82 and Cys93–Cys113 (yellow sticks), are indicated. The hairpin loops L1 and L2 and the N-terminus of cystatins are involved in the binding of papain-type cysteine proteases. (**C**) Superposition of C*α* traces of the mialostatin structure with five other cystatin structures including OmC2 from the soft tick *O. moubata* (PDB code 3L0R), iristatin from the hard tick *I. ricinus* (5O46), sialostatin L2 from the hard tick *I. scapularis* (3LH4), and representative vertebrate members of family 2 cystatins: chicken egg white (CEW) cystatin (1CEW) and human cystatin D (1RN7). The orientation of mialostatin is as in (**B**). Color coding of the structures and positions of the binding sites for papain-type cysteine proteases and legumains are indicated. (**D**) Structure-based sequence alignment of mialostatin with OmC2, iristatin, sialostatins L1 and L2, CEW cystatin, and human cystatin D. Residues identical to those of mialostatin are shaded grey. The secondary structural elements of mialostatin are depicted in magenta for β-strands and cyan for α-helices. The conserved disulfide bridges are indicated by the connecting black lines. Three regions involved in the interaction between cystatins and papain-type cysteine proteases are boxed in green and labeled (the region size was selected based on the predominant binding residues in the available complex structures); the consensus core residues are highlighted in red. The legumain binding site in CEW cystatin is highlighted in blue. Mature protein sequences were used in the alignment; residue numbering is according to mialostatin. (**E**) A comparison of the inhibitory potency of mialostatin with the other family 2 cystatins (shown in **D**) against various cysteine proteases including human papain-type cathepsins L to B (hCatL to hCatB) and mammalian legumains. IC_50_ values are presented [23,32,33,35,36,37] and displayed as a heat map (green scale); n.i.—not inhibited; x—no literature data are available.

Phylogenetic analysis clearly demonstrated that mialostatin belongs to the cystatin superfamily. According to the maximum likelihood method, the tick cystatin phylogenetic tree contained three separate prostriate clades (Figure 5A and Figure S1). As shown in the simplified tree in Figure 5A, mialostatin fell into a strongly supported group with four other cystatins from the genus *Ixodes*. This clade was distant from other clades, including the recently described iristatin [32] and previously characterized sialostatins L and L2 [33,34]. In general, tick cystatins cluster into several clades specific for either prostriate, metastriate, or argasid tick species, suggesting fast evolution of cystatin genes in ticks. The full phylogenetic tree presented in Figure S1 shows strong bootstrap support for smaller clades, but the topology is less clear closer to the root of the tree. The analysis shows mialostatin as a new distinct *Ixodes* cystatin consistent with its presumed major role in the midgut, as most previously characterized cystatins from *Ixodes* spp. are of salivary origin.

The crystal structure of mialostatin was determined by molecular replacement using the structure of the tick cystatin OmC2 as a search model and refined using data to 1.55 Å resolution (Table S2). The hexagonal prism crystal form contained two molecules in the asymmetric unit with a solvent content of about 57%. All protein residues could be modeled into a well-defined electron density map with the exception of the first nine residues, which formed a flexible N-terminus (Ser1 to Gly9), and the last two C-terminal residues (Asn118, Val119) of chain A. The final model consisted of two mialostatin molecules, chains A and B, containing 108 and 110 residues, respectively. The root-mean-square deviation (RMSD) for superposition of the Cα atoms of the two chains was 0.14 Å, a low value within the range observed for different crystal structures of identical proteins. 

Figure 5B shows the overall structure of mialostatin. The molecule adopts a typical cystatin fold (so called ‘hot dog’ fold [38]) characterized by a five-stranded twisted antiparallel β-sheet wrapped around a central α-helix. Mialostatin contains two conserved disulfide bridges connecting Cys69 with Cys82 and Cys93 with Cys113. Structural comparison and sequence alignment with other known cystatin structures clearly demonstrated that mialostatin belongs to family 2 of the cystatin superfamily (Figure 5C,D). The closest structural homolog of mialostatin was the salivary/gut cystatin OmC2 from the soft tick *O. moubata* [24] with the highest sequence identity (53%) and lowest RMSD for Cα (0.86 Å), followed by salivary homologs iristatin (41% identity, 1.56 Å RMSD) from the hard tick *I. ricinus* and sialostatins L1 (42%, 2.09 Å) and L2 (40%, 2.50 Å) from the hard tick *I. scapularis* [32,33]. Lower structural similarity was found with vertebrate members, namely human cystatin D (35%, 4.80 Å) and chicken egg white (CEW) cystatin (23%, 3.80 Å) (Figure 5D) [39,40].

The interaction between family 2 cystatins and papain-type cysteine proteases is mediated by three regions, the N-terminal segment and two hairpin loops L1 and L2, which form a tripartite wedge-shaped edge that binds to the enzyme active site cleft (Figure 5B,C) [40,41,42]. In mialostatin, the first part of the binding site is formed by the N-terminal segment around Gly10, which is the first visible residue in the electron density map. The conserved pair of glycines (Gly9, Gly10) provide conformational flexibility to the N-terminal segment to adopt an optimal conformation for target binding. The L1 loop (between β1 and β2) of mialostatin exposes the segment Gln51-Ile52-Val53-Ala54-Gly55 corresponding to the critical binding motif Gln-Xaa-Val-Xaa-Gly conserved in cystatins (Figure 5B). The L2 loop (between β3 and β4) is characterized in mialostatin and other cystatins, except sialostatins, by the presence of a conservative Pro101-Trp102 segment. To conclude, the structural analysis of mialostatin demonstrated a functionally competent reactive site against papain-type cysteine proteases. The binding motif for legumain-type cysteine proteases, which has been characterized in several cystatins (e.g., CEW cystatin), was absent in mialostatin (Figure 5C,D), consistent with the fact that mialostatin and other tick cystatins do not suppress legumain activity (Table 1, Figure 5E).

The inhibitory selectivity of the structurally analyzed cystatins is illustrated in Figure 5E. Mialostatin and OmC2 represent broad-spectrum inhibitors of papain-type cathepsins and are the most versatile in terms of their interactions of the analyzed tick cystatins. However, the other tick homologs displayed a distinct selectivity profile limited to effective inhibition of only some cathepsins. This may reflect structural changes in the conserved motifs on the L1 and L2 loops of iristatin and sialostatins, respectively, and in their N-terminal sequence potentially clashing with the partially occluded active sites of exopeptidases such as cathepsins B or H. Conversely, binding events to, for example, cathepsins B and C, can be supported by the electrostatic interactions formed by a positively charged basic patch (residues 12, 20, 106, 107) located at the reactive site of mialostatin and OmC2.

## 3. Discussion

*Ixodes ricinus* has previously been used as a model tick species to investigate and describe the complex intestinal digestive proteolytic mechanisms occurring in hematophagous arthropods. Blood proteins have been shown to be processed intracellularly by a multienzyme network of cysteine and aspartic proteases, with major involvement of cysteine cathepsin-type proteases from the CA clan [6]. However, previous studies have not investigated the regulation of digestive proteolysis, including the control mechanisms that protect the gut epithelium from excessive proteolysis and potential cell damage. Cystatins, naturally occurring cysteine protease inhibitors, are among the primary molecules of interest in the *I. ricinus* anti-proteolytic system, as they have been previously proposed to interact with digestive proteases in several other tick species [26,43,44].

In this study, we identified mialostatin as the first gut-associated cystatin to be identified in *I. ricinus* and present its comprehensive functional and structural characterization. Mialostatin was a potent inhibitor of *I. ricinus* digestive cysteine proteases of clan CA, covering both exopeptidases cathepsins B and C and endopeptidases cathepsins L1 and L3 (named IrCB, IrCC, IrCL1, and IrCL3, respectively). Its broad inhibitory selectivity is in clear contrast with *Ixodes* salivary cystatins such as sialostatins L1, L2, and iristatin, which have much narrower selectivity and mainly target endopeptidases [32,33,34]. On the other hand, similar broad anti-protease activity has been reported for OmC2 and partially also for OmC1 [23], cystatins present in the midgut of *O. moubata* soft ticks, or rBrBmcys2b from *Rhipicephalus microplus* [26] hard ticks. The 3D structural analysis identified mialostatin as a close homolog of OmC2 and provided a structural explanation for its binding selectivity through comparison of the architecture of the reactive site of mialostatin with other publicly available tick cystatin structures. Specifically, we highlight a combination of structural changes in three segments forming a tripartite wedge on mialostatin and OmC2 that slots into the cathepsin active site cleft. Based on structure-activity relationships and phylogenetic data, we propose that well-characterized mialostatin and OmC2 represent a new evolutionary subgroup of tick gut-associated cystatins that differ from salivary cystatins modulating host immune responses. Functional diversification of the cystatin superfamily is described in vertebrates [45]. It is likely that similar process occurs in ticks due to fast evolution of secreted proteins, therefore the phylogenetic tree reflects both localization and function of the cystatins. It is interesting to note that OmC2 also exhibits immunomodulatory properties, which correlate with its dual expression pattern in both the salivary glands and gut of *O. moubata* ticks, while OmC1 and mialostatin are expressed predominantly in tick midguts [23].

The biological role(s) of mialostatin in the tick gut can be inferred in the context of tick feeding behavior and associated physiological processes. Adult *I. ricinus* females engorge an enormous amount of host blood that exceeds the weight of the unfed tick more than a hundred-fold. The current model of the multienzyme digestive protease network responsible for blood protein processing is based mainly on investigations of the well-developed digestive midgut cells occurring in partially engorged *I. ricinus* females at the end of the slow feeding period at day 6–7 [13]. This period is followed by a rapid engorgement phase lasting 12–24 h, which accounts for about two-thirds of the total blood volume ingested before detachment from the host. Most blood proteins are used for vitellogenesis and massive egg production during several weeks off-host [5,46]. The molecular mechanisms underlying the associated protein turnover and long-term blood meal storage in the tick gut lumen remain unexplored, mainly due to technical limitations in studying fully fed females. Nevertheless, advances in the field and initial results led to the hypothesis that off-host digestion may include extracellular proteolysis of blood proteins in the gut lumen, which supports or replaces intracellular digestive proteolysis in the gut epithelium [47]. Despite the broad biochemical selectivity of mialostatin, its biological selectivity is limited due to compartmentalization in tick midgut cells. Our immunohistochemistry results demonstrated that mialostatin is localized to the same population of intracellular vesicles as IrCL3 on the 11th day post detachment, suggesting that mialostatin predominantly targets IrCL3. Mialostatin is stored in these vesicles in some cells even during tick feeding. Forming an inhibitory complex between mialostatin and IrCL3 might be relevant for intracellular trafficking of enzymatically inactive IrCL3 in tick gut cells. The localization of the mialostatin-IrCL3 complex to the surface of the large dense granules two weeks post detachment is probably associated with an excretion/secretion mechanism allowing translocation of the complex to the gut lumen. We hypothesize that IrCL3 might partially restore its proteolytic activity in the diluted contents of the gut lumen, where mialostatin can competitively interact with other secreted cysteine cathepsins including IrCL1 as the strongest mialostatin binder. This would enable cathepsin-mediated luminal proteolysis of blood proteins or the generation of antimicrobial peptides under general mialostatin control [48]. Luminal IrCL3 might also act as an anti-coagulation factor, as recently reported for a related *R. microplus* cathepsin L [49].

In conclusion, mialostatin is the first gut-associated cystatin characterized from *I. ricinus* at the functional and structural levels. Mialostatin localized to both digestive cells and the gut lumen, where it targets cathepsin L isoforms and regulates their activity during trafficking and processing of host blood proteins. As components of gut-associated proteolytic pathways, mialostatin and homologous cystatins in other tick species represent potential vaccination antigens for novel anti-tick interventions targeting tick reproduction. The vaccination efficacy of proteins derived from the tick gut (“concealed” antigens) in controlling tick infestations has already been successfully demonstrated [50], and new candidate antigens are increasingly in demand to combat tick infestations and to limit the global spread of tick-borne diseases.

## 4. Materials and Methods

### 4.1. Ticks and Laboratory Animals

All animal experiments were carried out in accordance with the Animal Protection Law of the Czech Republic No. 246/1992 Sb., ethics approval No. 34/2018, and protocols approved by the responsible committee of the Institute of Parasitology, Biology Centre of the Czech Academy of Sciences. Male and female adult *I. ricinus* ticks were collected by flagging in a forest near České Budějovice in the Czech Republic and then kept in 95% humidity chambers under a 12 h light/dark cycle at room temperature. Female BALB/c mice were purchased from Velaz (Prague, Czech Republic). Mice were housed in individually ventilated cages maintained under a 12 h light/dark cycle. Mice were used at 8–12 weeks of age. Laboratory rabbits were purchased from RABBIT CZ a. s. (Trhový Štěpánov, Czech Republic) and housed individually in cages in the animal facility of the Institute of Parasitology. Guinea pigs were bred and housed in cages in the animal facility of the Institute of Parasitology. All mammals were fed a standard pellet diet and provided with water ad libitum.

### 4.2. Quantitative Real-Time PCR

Female *I. ricinus* ticks were fed on rabbits and allowed to mate with male ticks. Salivary glands, midguts, and ovaries from five ticks per time point were dissected on a petri dish under a drop of ice-cold DEPC-treated PBS. Total RNA was isolated from dissected tissue using the NucleoSpin RNA kit (Macherey-Nagel, Düren, Germany) and its quality checked by agarose gel electrophoresis before storing the RNA at −80 °C. cDNA was prepared from 500 ng of total RNA from independent biological triplicates using the Transcriptor High-Fidelity cDNA Synthesis Kit (Roche Applied Science, Penzberg, Germany). The cDNAs served as templates for subsequent quantitative expression analyses of mialostatin transcription by qRT-PCR. Samples were analysed with a LightCycler 480 (Roche Applied Science, Penzberg, Germany) using FastStart Universal SYBR Green Master Mix (Roche Applied Science, Penzberg, Germany). Reaction conditions over 50 cycles were as follows: denaturation, 95 °C/10 s; annealing, 60 °C/10 s; extension, 72 °C/10 s. Relative expression values were standardized to a reference gene, *I. ricinus* elongation factor 1 (*ef1*; GenBank: GU074828) [51,52,53], and normalized to the sample with the highest level of expression. The primers sequences for mialostatin and *ef1* RT-PCR are shown in Table S3.

### 4.3. Mialostatin Cloning, Expression, Refolding, and Purification and Antibody Production

The full cDNA sequence of the gene encoding mialostatin was amplified using primers designed based on the GFVZ01041806.1 [28] transcript from NCBI GenBank. The primer sequences used for the final cloning of mialostatin are presented in Table S3. A pool of *I. ricinus* cDNA prepared from the salivary glands of female ticks fed for three and six days on rabbits was used as a template. The 372 base pair DNA fragment encoding mialostatin without a signal peptide and with an inserted ATG codon was cloned into a pET-17b vector (Novagen, Darmstadt, Germany) and transformed into *Escherichia coli* strain BL21(DE3)pLysS (Novagen) for expression. Bacterial cultures were grown in LB medium with 100 µg/mL ampicillin and 34 µg/mL chloramphenicol to an OD600 of 0.8, when protein expression was induced by the addition of isopropyl 1-thio-β-D-galactopyranoside to a final concentration of 1 mM. Cultures were harvested after 2 h of incubation at 37 °C at 200 rpm shaking speed. Isolated inclusion bodies were dissolved in 6 M guanidine hydrochloride, 20 mM Tris, and 10 mM DTT, pH 8 for 1 h followed by centrifugation (10 min, 10,000× *g*) to remove undissolved impurities. Refolding was performed by rapid dilution in 160 × excess of 20 mM Tris and 300 mM NaCl, pH 8.5. The resulting refolded protein was purified by HiLoad Superdex 200 26/60 gel filtration chromatography and HiPrep Q FF 16/10 ion exchange chromatography. Endotoxin was removed using a detergent-based method. Purified recombinant mialostatin was used to raise antibodies in a mouse and rabbit as described previously [54,55]. The immunoglobulin (Ig) fraction of rabbit serum was obtained by caprylic acid precipitation of serum proteins as described previously [56]. Hybridoma cells were raised by fusing splenocytes from immunized mice and mouse myeloma SP 2/0-Ag14 cells. Monoclonal antibodies were produced in cell culture following the previously described protocol [55].

### 4.4. Preparation of Tick Gut Samples

*I. ricinus* midguts were dissected from female *I. ricinus* fed on laboratory guinea pigs (samples for proteolysis analysis and Western blotting) or from females’ membrane fed on erythrocyte-depleted blood serum (samples for mass spectrometry analysis) [5]. The gut contents were carefully removed without disrupting the epithelium, and the gut tissue was washed in phosphate buffered saline (PBS). For mass spectrometry analysis, the gut contents were processed as described previously [6]. Gut tissue extract (150 mg protein/mL) was prepared by homogenization of the pooled gut tissue in 0.1 M Na acetate pH 4.5, 1% CHAPS on ice. The extract was cleared by centrifugation (16,000× *g*, 10 min, 4 °C), filtered through Ultrafree MC 0.22 µm (Millipore, Bedford, MA, USA), and stored at −80 °C.

### 4.5. Protease Inhibition Assays

Inhibition measurements were performed in triplicate in 96-well microplates (100 µL assay volume) at 37 °C. Recombinant mialostatin was preincubated with protease for 10 min followed by the addition of specific fluorogenic substrate (see Section 4.5.1, Section 4.5.2 and Section 4.5.3). The kinetics of product release were continuously monitored using an Infinite M1000 (Tecan, Männedorf, Switzerland) microplate fluorescence reader at 360 nm excitation and 465 nm emission wavelengths (for AMC-containing substrates) or at 320 nm excitation and 420 nm emission wavelengths (for Abz-containing substrate). IC_50_ values were determined from residual velocities using dose-response plots; nonlinear regression was fitted using GraFit software (Erithacus, East Grinstead, UK).

#### 4.5.1. Inhibition of Proteases in Tick Gut Homogenates

To prevent interference of non-target proteases, homogenates (80 ng) were treated with specific low molecular weight inhibitors (final assay concentrations are indicated) including 1 µM pepstatin and 1 mM EDTA (against aspartic proteases and metalloproteases; all assays), 1 µM E-64 (against cathepsins L/B; cathepsin C assay), 1 µM CA-074 (against cathepsin B; cathepsin L assay), and 1 µM Z–Phe–Phe–DMK (against cathepsin L; cathepsin B assay) [6]. The assay substrates and buffers were as follows: 20 µM Z–Phe–Arg–AMC substrate and 0.1 M Na acetate pH 4.5 or 5.0 in the cathepsin L and L/B assays, respectively; 20 µM Z–Arg–Arg–AMC substrate and 0.1 M MES pH 6.5 in the cathepsin B assay; 20 µM Gly–Arg–AMC substrate in 0.1 M Na acetate pH 5.5, 25 mM NaCl in the cathepsin C assay; all assay buffers contained 2.5 mM DTT and 0.1% PEG 1500.

#### 4.5.2. Inhibition Assays of Recombinant Tick Proteases

The assay conditions for individual proteases were as follows: 1.2 nM IrCD1 and 20 μM Abz–Lys–Pro–Ala–Glu–Phe–Nph–Ala–Leu substrate in 0.1 M Na acetate pH 4.0; 1.25 nM IrAE and 20 µM Z–Ala–Ala–Asn–AMC substrate in 0.1 M MES pH 5.0, 2.5 mM DTT, 1 µM E-64; 0.1 nM IrCL1 or 20 pM IrCL3 and 20 µM Z–Phe–Arg–AMC substrate in 0.1 M Na acetate pH 4.5, 2.5 mM DTT; all assay buffers contained 0.1% PEG 1500. The tick proteases were prepared as described elsewhere [7,9,10,57,58].

#### 4.5.3. Inhibition Assays of Human Proteases

Inhibition assays were performed following the same protocol used in our previous publications [24,32]. The assay conditions for individual proteases were as follows: 35 pM cathepsin B or 33 pM cathepsin L or 5 nM cathepsin K and 250 µM Z–Leu–Arg–AMC substrate in 0.1 M Na acetate pH 5.5, 0.1 M NaCl; 350 pM cathepsin S and 250 µM Z–Val–Val–Arg–AMC substrate in the same buffer; 0.5 nM cathepsin C and 250 µM Gly-Arg-AMC substrate in the same buffer; 20 nM cathepsin H and 40 µM Z–Leu–Arg–AMC substrate in 0.1 M Na/K phosphate pH 6.8; all assay buffers contained 1 mM EDTA, 2.5 mM DTT, and 0.01% Triton X-100. The human proteases were purchased from Merck (Kenilworth, NJ, USA) and Biomol (Hamburg, Germany).

### 4.6. Protein Digestion Assay

Digestion of 10 µg human serum albumin (Sigma Aldrich, St Louis, MO, USA), 5 µg bovine hemoglobin (Sigma Aldrich, St Louis, MO, USA), and 5 µg of mialostatin was performed with the tick gut tissue homogenate (0.4 µg protein) in 50 mM Na citrate pH 3.6, 2.5 mM DTT, in a total volume of 100 µL for 16 h at 26 °C. In the albumin and hemoglobin digestion assays, the homogenate was preincubated (15 min) in the same buffer with non-cysteine protease inhibitors: 1 µM pepstatin, 100 µM Pefablock, and 1 mM EDTA. The albumin digest was resolved with Laemmli SDS-PAGE gels (15%) and the hemoglobin and mialostatin digests by Tricine-SDS-PAGE gels (16% T/6% C) containing 6 M urea [59]. Electrophoresis was performed under reducing conditions, and protein was stained with Coomassie Blue G250.

### 4.7. Reducing SDS-PAGE and Western Blotting

Tick tissue homogenates were separated by reducing SDS-PAGE using 4–20% Mini-PROTEAN^®^ TGX™ Precast Protein Gels (Bio-Rad Laboratories, Hercules, CA, USA). Separated protein loads were visualized using TGX stain-free chemistry in the ChemiDoc MP imager (Bio-Rad, Hercules, CA, USA). After protein load documentation, separated proteins were electro-transferred from the gel onto an Immun-Blot^®^ LF PVDF membrane using the Trans-Blot Turbo system (Bio-Rad, Hercules, CA, USA). Prior to Western blot analyses, membranes were blocked with 3% non-fat milk in PBS with 0.05% Tween 20 (PBS-Tween) for 1 h at room temperature. Blocked membranes were incubated with the rabbit Ig fraction of α-IrCL1 or α-IrCL3 polyclonal sera diluted 1:1000 in PBS-Tween containing 1% milk. Goat anti-rabbit IgG Alexa 488-labeled antibody (1:1000, Thermo Fisher Scientific, Waltham, MA, USA) was used as a secondary antibody. For mialostatin detection, α-mialostatin monoclonal antibody (1:30) diluted in in PBS-Tween containing 1% milk and the goat anti-mouse Alexa 546-labeled antibody (1:1000, Thermo Fisher Scientific, Waltham, MA, USA) were used. In between individual steps of the whole procedure, membranes were washed 3 × 5 min in PBS-Tween on a rotating shaker platform at room temperature. Labeling with primary antibodies was performed on a rotating shaker platform at 4 °C overnight. Labeling with secondary antibodies was performed on a rotating shaker platform at room temperature for 1 h. Fluorescent signals were again visualized using the ChemiDoc MP imager and analyzed using Image Lab Software (Bio-Rad, Hercules, CA, USA).

### 4.8. Immunohistochemistry

Samples of *I. ricinus* gut tissues were prepared as described previously [11]. Briefly, the gut was dissected from adult females at specific days of feeding on the host and days post-attachment and fixed in 4% formaldehyde and 0.1% glutaraldehyde solution, washed with PBS, dehydrated using ascending ethanol dilutions, then infiltrated with LR White resin (London Resin Company, Stansted, UK) and polymerized. Semi-thin sections (0.5 µm) were blocked with 1% BSA and 1% milk in PBS-Tween (0.3% (*v*/*v*) Tween 20) for 45 min. Immunohistochemical double-staining was performed gradually, with the initial antibody labeling of the respective intestinal protease (*I. ricinus* cathepsin L1 IrCL1 [9]; cathepsin L3 IrCL3; cathepsin B IrCB [11]) subsequently followed with immunolabeling of mialostatin. First, semi-thin tick gut tissue sections were blocked with blocking solution (1% BSA, 1% milk solution in PBS-Tween) for 45 min at room temperature. For protease immunostaining, sections were first labeled (4 °C overnight) with primary antibodies: (i) rabbit α-IrCL1 affinity-purified polyclonal serum diluted 1:5 in PBS-Tween; (ii) rabbit α-IrCB affinity-purified polyclonal serum diluted 1:5 in PBS-Tween; (iii) isolated Ig fraction of α-IrCL3 polyclonal serum diluted 1:5 (IrCL1) in PBS-Tween. After washing 3 × 5 min in PBS-Tween, sections were subsequently labeled with Alexa Fluor^®^ 647 goat α-rabbit secondary antibody (diluted 1:500 in PBS-Tween; Thermo Fisher Scientific, Waltham, MA, USA). Sections were subsequently used for mialostatin immunolabeling: sections were once again washed 3 × 5 min in PBS-Tween and incubated with mouse α-mialostatin monoclonal antibody diluted 1:50 in PBS-Tween. Incubation was performed in a humid chamber at room temperature for 90 min. Sections were once again washed (3 × 5 min in PBS-Tween) and incubated with secondary goat α-mouse Alexa Fluor^®^ 488 (Thermo Fisher Scientific, Waltham, MA, USA) diluted 1:500 in PBS-Tween for 1 h at room temperature. Finally, all sections were washed in PBS-Tween and counterstained with DAPI (4′,6′-diamidino-2-phenylindole; 2.5 μg/mL; Sigma Aldrich, St Louis, MO, USA) for 7 min, washed again with PBS-Tween, mounted in Fluoromount medium (Sigma Aldrich, St Louis, MO, USA), and examined with the IX83 confocal microscope (Olympus, Tokyo, Japan). Images were processed with FluoView FV3000 software (Olympus, Tokyo, Japan).

### 4.9. Evolutionary Analysis by the Maximum Likelihood Method

The evolutionary history was inferred using the maximum likelihood method and JTT matrix-based model [60]. The bootstrap consensus tree inferred from 1000 replicates was taken to represent the evolutionary history of the taxa analyzed [61]. Branches corresponding to partitions reproduced in less than 20% bootstrap replicates were collapsed. The percentage of replicate trees in which the associated taxa clustered together in the bootstrap test (1000 replicates) are shown next to the branches [61]. Initial tree(s) for the heuristic search were obtained automatically by applying Neighbor-Join and BioNJ algorithms to a matrix of pairwise distances estimated using the JTT model and then selecting the topology with the superior log-likelihood value. This analysis involved 71 amino acid sequences. There were 108 positions in the final dataset. Evolutionary analyses were conducted in MEGA X [62].

### 4.10. Crystallization and Data Collection

Screening for crystallization conditions was performed using the JCSG-plus kit (Molecular Dimensions, Sheffield, UK) by the sitting drop vapor diffusion technique. Preliminary crystals of mialostatin were obtained in 0.1 M citric acid pH 3.5, 0.8 M ammonium sulfate. Optimal crystals were prepared at 18 °C using the hanging drop vapor diffusion technique in 15-well NeXtal plates (Qiagen, Hilden, Germany). The crystallization drop consisted of 2 μL of the mialostatin protein solution (12.5 mg/mL in 10 mM Tris buffer, pH 8.0) and 1 μL of the precipitant solution equilibrated over a reservoir containing 300 µL precipitant solution (0.1 M citric acid pH 4.0, 0.8 M ammonium sulfate). Crystals shaped as hexagonal prisms reached their final size of 0.6 × 0.3 × 0.3 mm within 1 month. For data collection, crystals were soaked in reservoir solution supplemented with 20% glycerol and flash cooled in liquid nitrogen. Diffraction data at 100 K were collected using a BL14.1 beamline operated by the Helmholtz-Zentrum Berlin (HZB) at the BESSY II electron storage ring (Berlin-Adlershof, Germany) [63] and processed using the XDS suite of programs [64]. Crystals exhibited the symmetry of space group P6222 and contained two molecules in the asymmetric unit. Crystal parameters and data collection statistics are shown in Table S2.

### 4.11. Structure Determination

The phase problem was solved by molecular replacement using Molrep [65] from the CCP4 package [66]. The search model was derived from the structure of cystatin OmC2 (PDB code 3L0R) [24] sharing 53% sequence identity with mialostatin. Model refinement was carried out using REFMAC 5.8 [66] from the CCP4 package with 5% of the reflections reserved for cross-validation. Manual building and addition of water molecules was performed using Coot [67]. The quality of the final model was validated with Molprobity [68]. Final refinement statistics are given in Supporting Information Table S2. Figures showing structural representations were prepared with the PyMOL Molecular Graphics System (Schrödinger, New York, NY, USA). Atomic coordinates and structure factors were deposited in the PDB under accession code 6ZTK.

### 4.12. Statistical Analysis

All experiments were performed in biological triplicate. Data are presented as mean ± standard error of mean (SEM) in all graphs. Student’s t-test or one-way ANOVA were used to calculate statistical differences between two or more groups, respectively. Statistically significant results are marked: * *p* ≤ 0.05; ** *p* ≤ 0.01; *** *p* ≤ 0.001; **** *p* ≤ 0.0001.

## Figures and Tables

**Table 1:** Inhibitory effect of mialostatin on the activity of tick and human proteases. The inhibitory potency of mialostatin was determined against: (i) native cysteine cathepsins present in *I. ricinus* gut tissue extract using protease-specific assays (left panel); (ii) selected digestive proteases of *I. ricinus* prepared as recombinant proteins (middle panel); and (iii) a representative set of human cysteine cathepsins (right panel). The IC_50_ values (mean values ± SE) were measured by kinetic activity assays using specific fluorogenic peptide substrates (for details, see Methods).

Inhibition of *I. ricinus* Midgut Homogenate		Inhibition of Recombinant Digestive *I. ricinus* Proteases		Inhibition of Human Cysteine Cathepsins	
Targeted Activity	IC_50_ (nM)	Proteases	IC_50_ (nM)	Protease	IC_50_ (nM)
Cathepsin L	0.18 ± 0.02	*Ir*-cathepsin L1 (IrCL1)	0.071 ± 0.01	*Hs*-cathepsin L	0.38 ± 0.03
Cathepsins L and B	3.1 ± 0.4	*Ir*-cathepsin L3 (IrCL3)	0.39 ± 0.18	*Hs*-cathepsin C	2.1 ± 0.8
Cathepsin B	12.1 ± 1.5	*Ir*-legumain (IrAE)	n.i.	*Hs*-cathepsin S	2.2 ± 0.4
Cathepsin C	91.7 ± 5.5	*Ir*-cathepsin D1 (IrCD1)	n.i.	*Hs*-cathepsin B	9.0 ± 0.3
				*Hs*-cathepsin K	9.7 ± 1.3
				*Hs*-cathepsin H	24.0 ± 3.5

Abbreviation: n.i.—no significant inhibition at 10 μM mialostatin concentration.

## Data Availability

All data are either contained within the manuscript and supporting information or available from the corresponding author on reasonable request.

## Supplementary Materials

The following are available online at https://www.mdpi.com/article/10.3390/ijms22105371/s1, Figure S1: The phylogenetic tree of 71 cystatins from both Ixodidae and Argasidae tick species, Figure S2: Detection of mialostatin in midgut of blood fed ticks, Figure S3: Detection of mialostatin in midgut of serum fed ticks, Figure S4: Detection of *I. ricinus* cathepsins L in midgut of blood fed ticks, Table S1: Identification of mialostatin by mass spectrometry, Table S2: X-ray data collection and refinement statistics, Table S3: Primer sequences.
