# Supplementary material for: Mialostatin, a Novel Midgut Cystatin from Ixodes ricinus Ticks: Crystal Structure and Regulation of Host Blood Digestion
Source: Int J Mol Sci. 2021 May 20;22(10):5371. doi: 10.3390/ijms22105371 (PMC8161381; doi:10.3390/ijms22105371)
Supplement: Supplementary file 1 [file ijms-22-05371-s001.zip › ijms-1207856-supplementary.pdf]

Supplementary Materials

Table S1. Identification of Mialostatin by mass spectrometry

Equal protein amounts (2.5 µg) of gut tissue and gut lumen contents of *I. ricinus* adult females were reduced with DTT, alkylated with iodacetamide, and digested in parallel with trypsin or chymotrypsin. Peptide digests were reconstituted in 0.1% formic acid and separated on a PepMap RSLC C18 analytical column (2 µm, 15 cm × 75 µm, Thermo Fisher Scientific) using a linear gradient of acetonitrile. The LC-MS/MS analysis was performed on the UltiMate 3000 RSLCnano system (Thermo Fisher Scientific) coupled to Orbitrap Fusion Lumos (Thermo Fisher Scientific). MS scans were recorded from 350 to 2000 m/z in orbitrap; in MS/MS mode the fragmentation spectra were acquired within the mass range of 100–2000 m/z. Proteome Discoverer 2.4 (Thermo Fisher Scientific) was used for protein identification against the UniProtKB/Swiss-Prot and TrEMBL databases. Peptide coverage of the mature Mialostatin sequence at a mass accuracy of <5 p.p.m. is indicated.

Ticks	Sample	Sequence coverage (%)
Fully fed	Gut tissue	71
	Gut lumen content	48
5 Days after detachment	Gut tissue	49
	Gut lumen content	11

Table S2. X-ray data collection and refinement statistics

**Data collection**	
Space group	$P2_{2}22$
Cell dimensions	
a, b, c (Å)	81.11, 81.12, 162.33
α, β, γ (°)	90.00, 90.00, 120.00
Number of molecules in AU	2
Wavelength (Å)	0.918
Resolution range (Å)	42.9 - 1.55 (1.65 - 1.55)
Number of unique reflections	46381 (4528)
Redundancy/multiplicity	21.2 (21.5)
Completeness (%)	99.90 (99.25)
R_{merge}	0.084 (1.203)
Average I/s(I)	21.95 (2.00)
Wilson B ($Å^2$)	19.54
**Refinement**	
Resolution range (Å)	42.9 - 1.55 (1.65 - 1.55)
No. of reflections in working set	44052 (4528)
No. of reflections in test set	2328 (237)
R value (%)	17.76
R_{free} value (%)	20.83
RMSD bond length (Å)	0.017
RMSD angle (°)	1.94
Number of atoms in AU	2243
Number of protein atoms in AU	1809
Number of water molecules in AU	371
**Ramachandran plot**	
Residues in favored regions (%)	2.34
Residues in allowed regions (%)	97.66

Table S3. Primer sequences

Amplicon name	Forward primer 5´ - 3´	Reverse primer 5´ - 3´
Mialostatin cloning	CATATGATGGGGTCGGCGAGCA	CTCGAGCTAGACATCATTAGGA
Mialostatin RT-PCR	GAGGTGCAGACTCAGATTGTGG	GCATACAGCTTCAACCTTGTTTGTC
Ef1 RT-PCR	ACGAGGCTCTGACGGAAG	CACGACGCAACTCCTTCAC

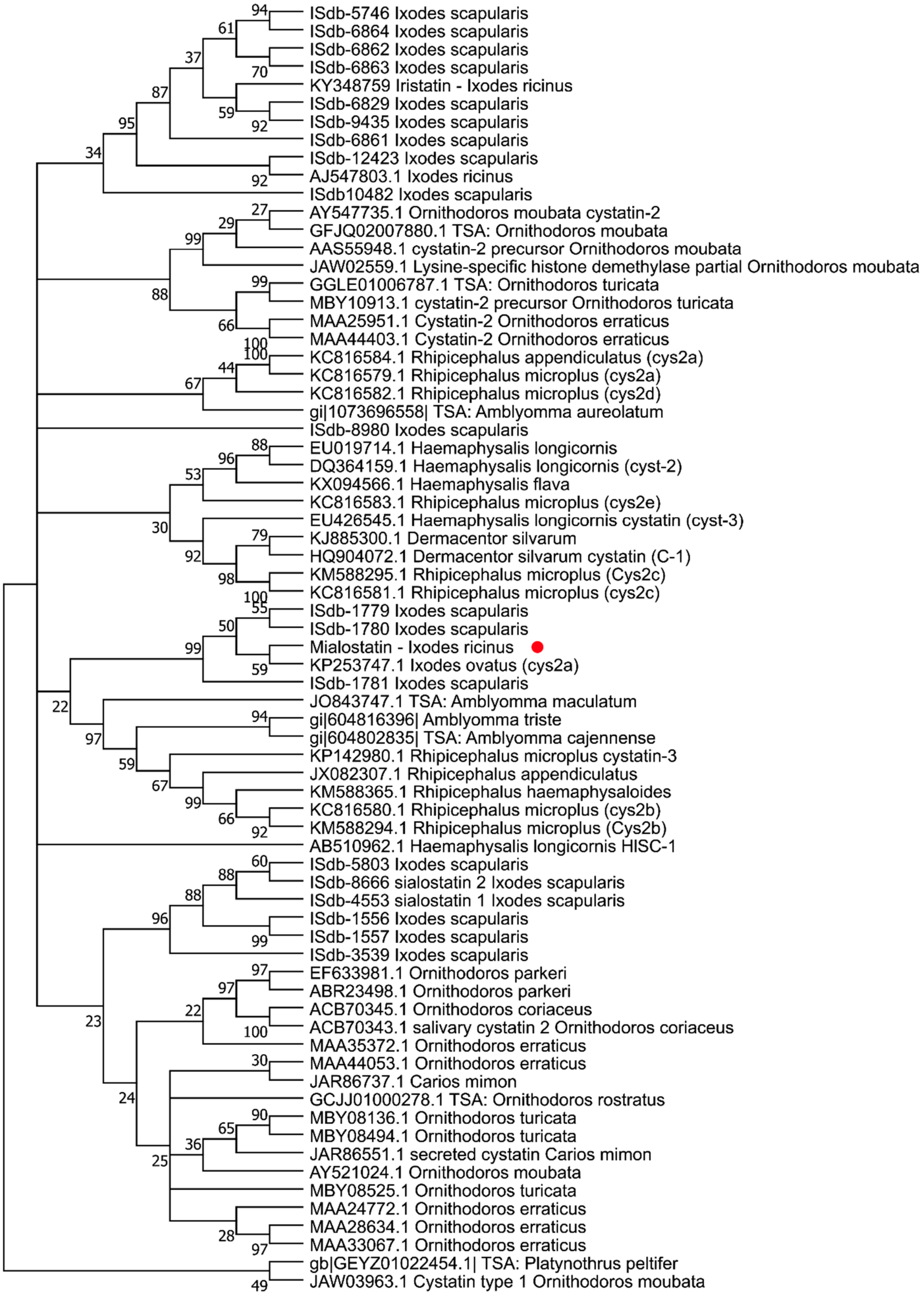

Figure S1: The phylogenetic tree of 71 cystatins from both Ixodidae and Argasidae tick species was prepared by using the maximum likelihood method and JTT matrix-based model (1). The bootstrap consensus tree is inferred from 1000 replicates (2). Branches corresponding to partitions reproduced in less than 20% bootstrap replicates are collapsed. The percentage of replicate trees in which the associated taxa clustered together in the

bootstrap test (1000 replicates) are shown next to the branches. Evolutionary analyses were conducted in MEGA X (3).

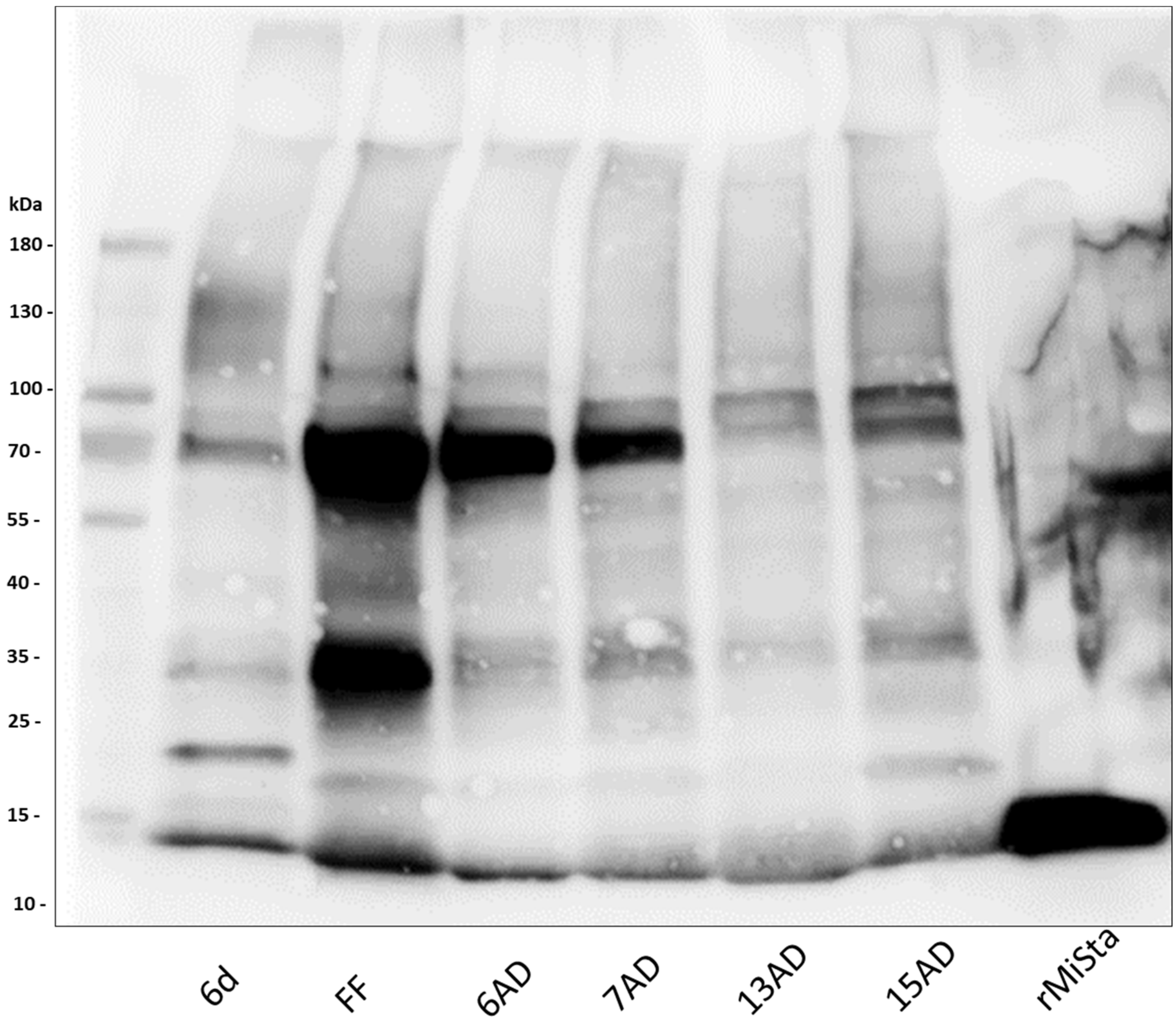

Figure S2: Detection of Mialostatin in midgut of blood fed ticks
Full view on Western blot presented in Figure 3A, left. Bands at higher molecular weight than 13kDa probably represent Mialostatin multimers or complexes.

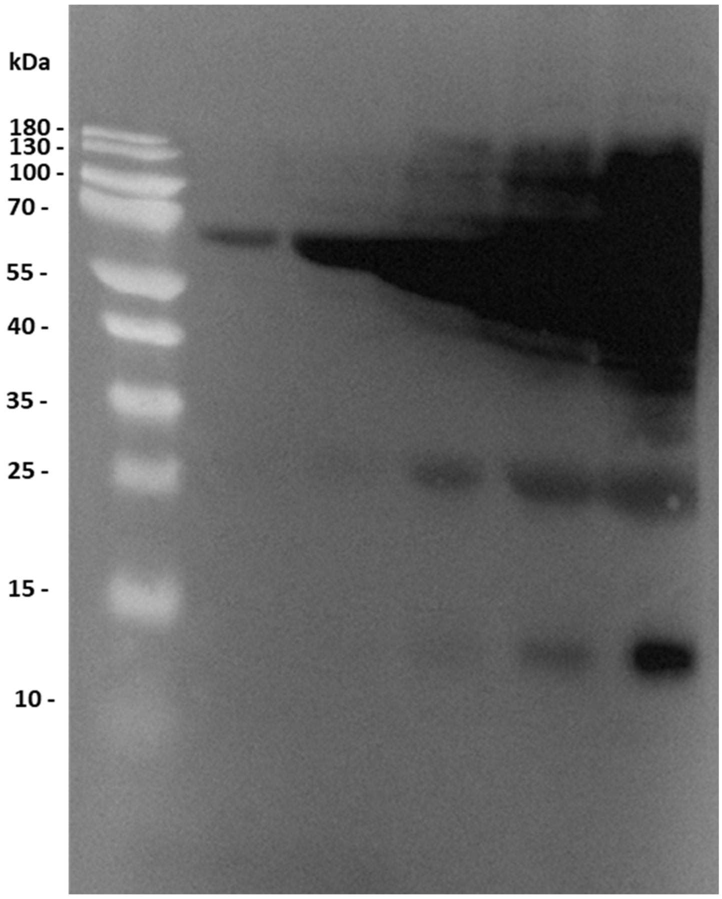

Figure S3: Detection of Mialostatin in midgut of serum fed ticks
Full view on Western blot presented in Figure 3A, right. Lanes differ in protein load and only lane in the right was used for Figure 3A. All samples represent luminal fluid collected from tick midgut after fully fed. Bands at higher molecular weight than 13kDa probably represent Mialostatin multimers or complexes.

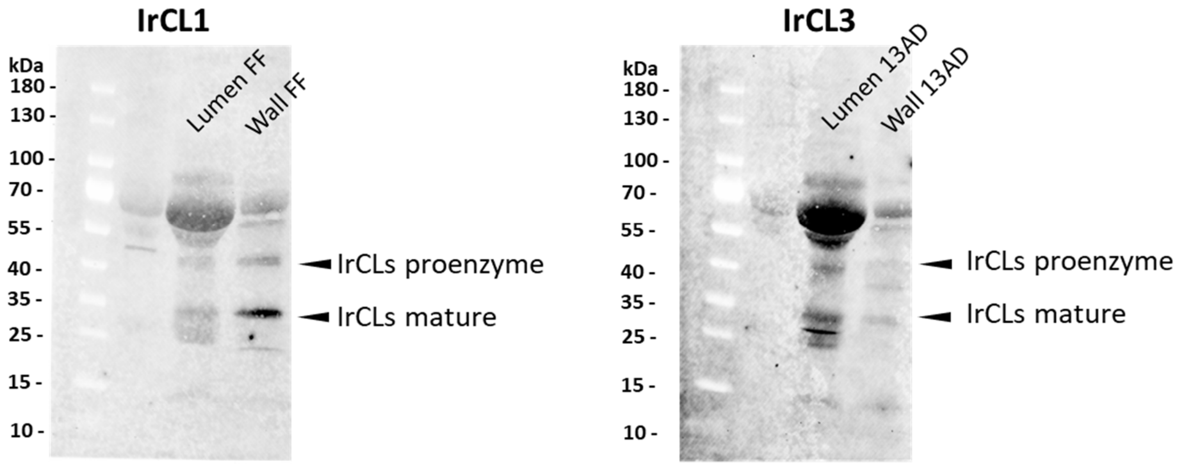

Figure S4: Detection of *I. ricinus* cathepsins L in midgut of blood fed ticks
Full view on Western blot presented in Figure 3B.

References

1. Jones, D. T., Taylor, W. R., and Thornton, J. M. (1992) The rapid generation of mutation data matrices from protein sequences. *Comput. Appl. Biosci.* **8**, 275-282
2. Felsenstein, J. (1985) Confidence Limits on Phylogenies: An Approach Using the Bootstrap. *Evolution* **39**, 783-791
3. Kumar, S., Stecher, G., Li, M., Knyaz, C., and Tamura, K. (2018) MEGA X: Molecular Evolutionary Genetics Analysis across Computing Platforms. *Mol. Biol. Evol.* **35**, 1547-1549
